# Histiocytic Sarcoma Diagnosed on Repeat Biopsy of Multifocal Extranodal Tumors

**DOI:** 10.70352/scrj.cr.25-0133

**Published:** 2025-05-29

**Authors:** Toshinari Ito, Yuya Iwata, Yoshimasa Akiba, Saki Ishiya, Toshiki Okasaka

**Affiliations:** Department of Thoracic Surgery, Aichi Koseiren Toyota Kosei Hospital, Toyota, Aichi, Japan

**Keywords:** histiocytic sarcoma, VATS, BRAF, biopsy

## Abstract

**INTRODUCTION:**

Histiocytic sarcoma (HS) is a rare malignant disease with a poor prognosis and unknown pathogenesis. In addition, this disease is difficult to diagnose due to the wide variety of diseases to be differentiated from it.

**CASE PRESENTATION:**

A 37-year-old woman with right-sided chest pain was referred to our hospital. Computed tomography (CT) revealed a 30-mm mass with osteolytic changes in the right eighth rib. Further examination revealed a rib mass, right renal mass, anterior mediastinal nodule, bilateral pulmonary nodules, and suprasellar nodule. A needle biopsy of the rib mass revealed granuloma with histiocytes. A subsequent needle biopsy of the right renal mass revealed similar findings without evidence of malignancy. The imaging findings suggested malignant disease; therefore, wedge resection of the upper lobe of the right lung and biopsy of the mediastinal mass were performed under general anesthesia. HS was diagnosed based on the immunostaining results.

**CONCLUSIONS:**

For the treatment of HS, early intervention is said to contribute to a prolonged prognosis. In order to provide appropriate therapy, HS should be included in the differential diagnosis for multifocal extranodal tumors.

## Abbreviations

CT
: computed tomography
FDG
: fluorodeoxyglucose
HS
: histiocytic sarcoma
PET
: positron emission tomography

## INTRODUCTION

Histiocytic sarcoma (HS), a rare malignant disease, originates from histiocytic cells in the lymph nodes and forms tumors in multiple organs.^1,2)^ The pathogenesis of HS is unknown and the prognosis is poor.^3)^ One of the problems in treating this disease is the difficulty of diagnosing it accurately. Herein, we report a case of HS that required multiple surgical biopsies to determine the definitive diagnosis.

## CASE PRESENTATION

**Fig. 1:**
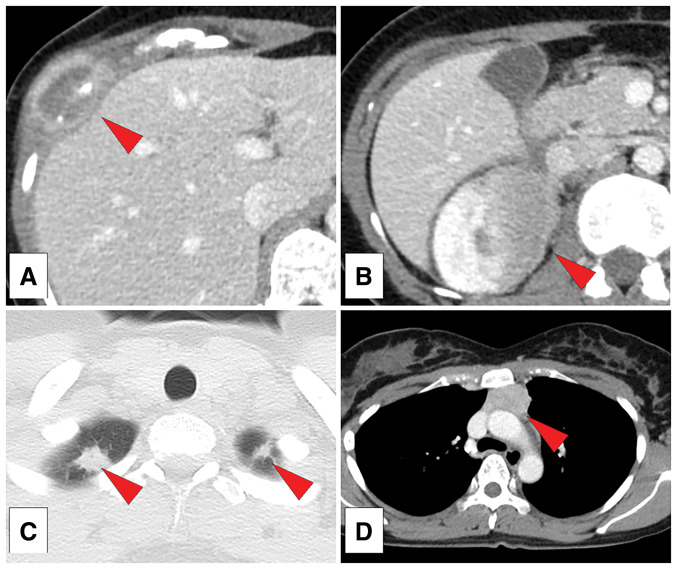
Computed tomography (CT) with contrast. (**A**) A 30-mm-wide mass with osteolytic changes in the right eighth rib. (**B**) A 42-mm-wide right renal mass. (**C**) Bilateral lung nodules with diameters of 13 mm and 6 mm. (**D**) A 34-mm-wide anterior mediastinal mass.

The patient was a 37-year-old woman without a relevant medical history, who was referred to our hospital because of right-sided chest pain. Chest computed tomography (CT) revealed a 30-mm mass with osteolytic changes in the right eighth rib (**Fig. 1A**).

**Fig. 2:**
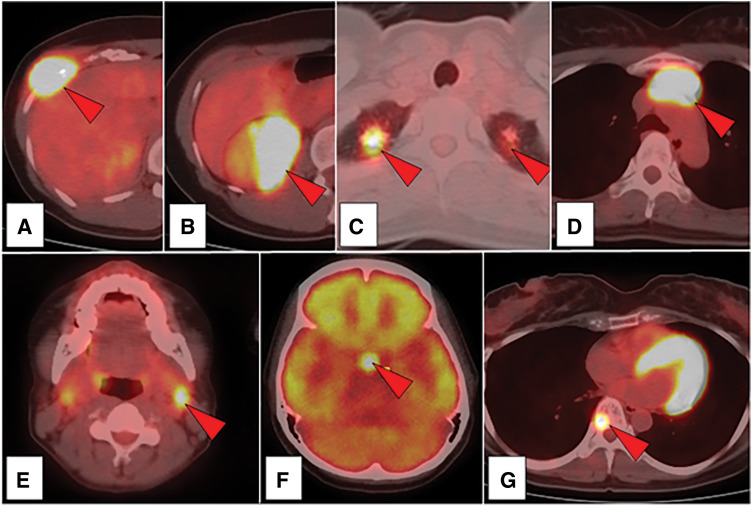
Fluorodeoxyglucose (FDG)-positron emission tomography (PET)/computed tomography (CT). High uptake of FDG is observed in the nodules (red arrows). (**A**) The right eighth rib. (**B**) The right kidney. (**C**) Bilateral lung nodules. (**D**) The anterior mediastinal nodule. (**E**) Left cervical lymph nodes. (**F**) Suprasellar nodule. (**G**) Eighth thoracic spine.

Whole-body CT revealed a rib mass, 42-mm right renal mass (**Fig. 1B**), 13-mm right upper lobe lung nodule, 6-mm left upper lobe lung nodule (**Fig. 1C**), and 34-mm anterior mediastinal mass (**Fig. 1D**). Positron emission tomography-CT showed fluorodeoxyglucose hyperaccumulation (**Fig. 2A**–**2D**) and hyperintensity in the left cervical lymph node (**Fig. 2E**), suprasellar node (**Fig. 2F**), and eighth thoracic spine (**Fig. 2G**). Blood biochemical tests revealed a normal complete blood count and normal tumor markers.

CT-guided biopsy of the right rib mass was performed under local anesthesia, resulting in the pathological diagnosis of a granulomatous lesion. Because of the possibility of an insufficient specimen, a CT-guided biopsy of the right renal mass was performed under local anesthesia. The pathological results indicated infiltration and aggregation of large cells, which were considered histiocytes, with fibrous connective tissue hyperplasia and partial sclerosis. Immunostaining results were negative for S-100 and CD1a and positive for CD163 and CD68, confirming the diagnosis of histiocytic lesions; however, malignant findings such as malignant lymphoma were not observed. The disease course suggested the possibility of malignant disease, and immunostaining results indicated HS and Erdheim–Chester's disease as differentials. To obtain more specimens, thoracoscopic wedge resection of the upper lobe of the right lung and biopsy of the mediastinal mass were performed under general anesthesia.

**Fig. 3:**
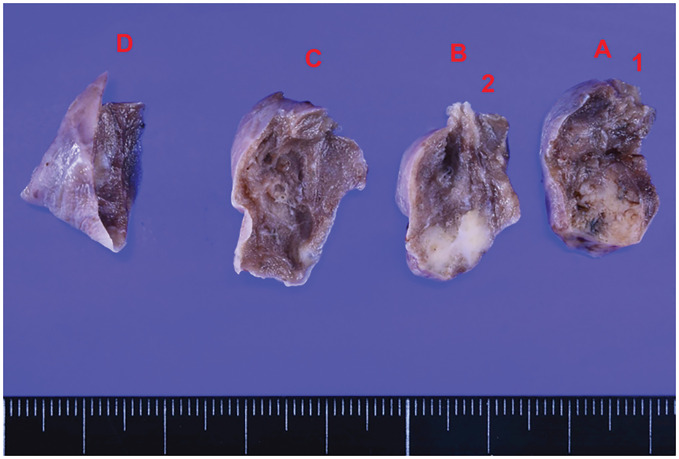
Specimen resected from the lung nodule. The pulmonary nodule is indurated, and the cut surface is white.

Because the disease course suggested the possibility of malignant disease, thoracoscopic wedge resection of the upper lobe of the right lung and biopsy of the mediastinal mass were performed under general anesthesia. The resected lung nodule was hard, and homogeneous white tissue was observed on the cut surface (**Fig. 3**).

**Fig. 4:**
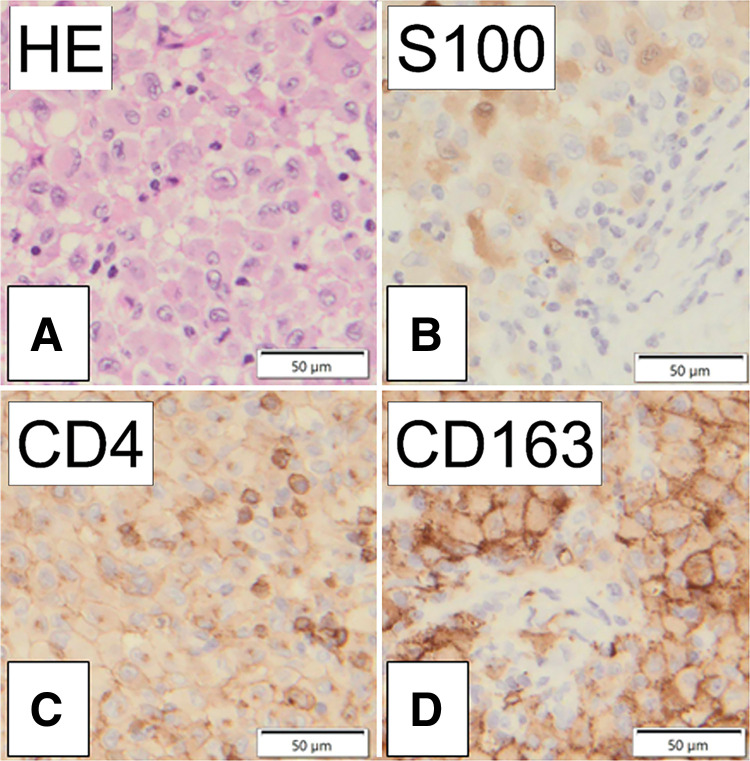
Results of the pathological and immunohistochemical evaluations. (**A**) Hematoxylin and eosin (HE) stain (×40) showing the proliferation of cells with large nuclei with prominent nipples and bubble spores. (**B**) Negative for S100. (**C**) Negative for CD4. (**D**) Positive for CD163.

Biopsies of the right lung nodule and mediastinal mass revealed similar results. Histopathological examination revealed a granular chromatin pattern with oval-to-irregular nuclei exhibiting indentations. Prominent nucleoli were eccentrically located, accompanied by abundant eosinophilic cytoplasm showing a foamy appearance. Immunostaining revealed negative for S-100, CD4, CD1a, AE1/AE3, CD30, BRAF V600E, and ALK and positive for CD163, CD68, CD30 (weak), CD3, and PAX5 (**Fig. 4**). Therefore, HS was established as the definitive diagnosis. The time from first examination to the final diagnosis was 118 days.

No standard treatment for HS exists. The FoundationOne cancer gene panel test (Foundation Medicine, Boston, MA, USA) was used to comprehensively search for gene mutations. The BRAF gene mutation was identified as an AGAP3::BRAF fusion; however, no drugs or clinical trials were suitable for this mutation. After initial radiotherapy for a suprasellar tumor and symptomatic right rib lesions, chemotherapy with the CHOP regimen (cyclophosphamide, doxorubicin, vincristine, and prednisolone), referencing standard protocols for lymphoma treatment, was initiated. Seven months after diagnosis and following six cycles of CHOP, a PET-CT scan revealed overall tumor shrinkage with reduced FDG uptake. However, a new osteolytic lesion was detected in the left sixth rib, leading to an assessment of progressive disease. Based on a second opinion, second-line chemotherapy with eribulin mesylate was initiated. Subsequent periodic imaging evaluations have shown partial response or stable disease. At present, 2 years and 6 months after initial diagnosis, treatment with eribulin mesylate continues.

## DISCUSSION

HS, which is a tumor derived from macrophages, is classified as one of the malignant histiocytosis in the five histiocytosis categories.^1,2)^ HS accounts for less than 1% of hematopoietic tumors and has a reported incidence of 0.17 per 1 million individuals; common sites of HS include the skin, lymph nodes, and the respiratory and nervous systems.^3)^ The most common site of HS is the hematopoietic tissue, such as lymph nodes. HS is associated with various systemic symptoms, such as fever, weight loss, and general malaise. However, when extranodal lesions develop in the gastrointestinal tract, spleen, soft tissues, or skin, systemic symptoms are rare; for cases such as rib tumors, localized pain is often the main symptom.

The differential diagnoses of HS are diverse; therefore, establishing a definitive diagnosis is difficult.^4–6)^ Our patient underwent biopsies three times that yielded positive findings for CD163 and CD68 and negative findings for lymphatic, epithelial cell, melanocyte, and dendritic cell lineage results, thus confirming the diagnosis of HS.

Patients with HS have a generally poor prognosis and overall survival ranging from 6 months to 2 years.^7,8)^ No standard treatment for HS has been established; therefore, chemotherapy similar to that used for malignant lymphoma is commonly administered.^9)^ Surgical resection may prolong the survival of patients with localized pathology; therefore, surgical treatment is sometimes the preferred treatment. However, responses to BRAF inhibitors in cases of major mutations of the BRAF gene^10,11)^ and responses to pembrolizumab in cases of high PD-L1 expression^12,13)^ have been reported.

One report of HS indicated that a median of two times tissue biopsies were required to confirm the diagnosis and that the median time from symptom onset to diagnosis was 2.5 months.^14)^ Although we performed two biopsies on our patient, the results did not indicate a definitive diagnosis. CT and PET were performed to investigate the cause of pain, and the results strongly suggested a malignant lesion; therefore, a surgical lung biopsy was performed under general anesthesia, resulting in establishing the definitive diagnosis of HS. In this patient, the rib mass was palpable from the body surface and considered minimally invasive, making it the most suitable site for the initial biopsy. However, since a definitive diagnosis could not be reached, a second biopsy was necessary. At that time, the renal tumor was the largest and could be targeted for needle biopsy under local anesthesia. The second biopsy, however, yielded similar results and did not lead to a definitive diagnosis. In retrospect, given that the initial biopsy suggested a tumor that was challenging to differentiate, it would have been more appropriate to perform lung resection under general anesthesia rather than repeating a needle biopsy to ensure adequate sample volume. When treating diseases such as HS, which is rare and difficult to diagnose histopathologically, knowledge of the suspected disease is necessary. Performing a timely and appropriate invasive surgical biopsy under general anesthesia, along with considering rare diseases in the differential diagnosis, can lead to an early definitive diagnosis and appropriate treatment.

## CONCLUSIONS

We encountered a rare case of HS that was difficult to diagnose. HS should be included in the differential diagnosis for multifocal extranodal tumors. Timely and appropriate surgical interventions, such as a pulmonary biopsy under general anesthesia, are important for the initiation of the optimal treatment at an early stage.

## DECLARATIONS

### Funding

There is no funding source.

### Authors’ contributions

TI was a major contributor in writing the manuscript.

YI, YA, SI, and TO analyzed and interpreted the patient data.

All authors read and approved the final manuscript.

### Availability of data and materials

The authors have full control of all primary data and agree to allow the journal to review their data if requested.

### Ethics approval and consent to participate

This study was performed in accordance with the ethical standards of the Helsinki Declaration of 1964 and all its subsequent revisions. As it is a retrospective case report, the review and consent of the Ethics Review Committee is not considered to require.

### Consent for publications

Informed consent was obtained from the patient to publish this case report and associated images.

### Competing interests

The authors declare that they have no competing interests.

## References

[1] Emile JF Abla O Fraitag S Revised classification of histiocytoses and neoplasms of the macrophage-dendritic cell lineages. Blood 2016; 127: 2672–81. 26966089 10.1182/blood-2016-01-690636 PMC5161007

[2] Emile JF Cohen-Aubart F Collin M Histiocytosis. Lancet 2021; 398: 157–70. 33901419 10.1016/S0140-6736(21)00311-1 PMC9364113

[3] Kommalapati A Tella SH Durkin M Histiocytic sarcoma: a population-based analysis of incidence, demographic disparities, and long-term outcomes. Blood 2018; 131: 265–8. 29183888 10.1182/blood-2017-10-812495 PMC5757688

[4] Hung YP Qian X. Histiocytic sarcoma. Arch Pathol Lab Med 2020; 144: 650–4. 31070934 10.5858/arpa.2018-0349-RS

[5] Kurimoto T Goto T Yasuda T Histiocytic sarcoma of the palate: a case report. Int J Oral Maxillofac Surg 2023; 52: 1225–9. 37643937 10.1016/j.ijom.2023.08.003

[6] Bang S Kim Y Chung MS Primary histiocytic sarcoma presenting as a breast mass: a case report. J Breast Cancer 2019; 22: 491–6. 31598348 10.4048/jbc.2019.22.e32 PMC6769387

[7] Ansari J Naqash AR Munker R Histiocytic sarcoma as a secondary malignancy: pathobiology, diagnosis, and treatment. Eur J Haematol 2016; 97: 9–16. 26990812 10.1111/ejh.12755

[8] Facchetti F Pileri SA Lorenzi L Histiocytic and dendritic cell neoplasms: what have we learnt by studying 67 cases. Virchows Arch 2017; 471: 467–89. 28695297 10.1007/s00428-017-2176-1

[9] Tsujimura H Miyaki T Yamada S Successful treatment of histiocytic sarcoma with induction chemotherapy consisting of dose-escalated CHOP plus etoposide and upfront consolidation auto-transplantation. Int J Hematol 2014; 100: 507–10. 25062797 10.1007/s12185-014-1630-y

[10] Go H Jeon YK Huh J Frequent detection of BRAF^V600E^ mutations in histiocytic and dendritic cell neoplasms. Histopathology 2014; 65: 261–72. 24720374 10.1111/his.12416

[11] Zanwar S Abeykoon JP Dasari S Clinical and therapeutic implications of BRAF fusions in histiocytic disorders. Blood Cancer J 2022; 12: 97. 35764604 10.1038/s41408-022-00693-7 PMC9240055

[12] Gao J Li M Liu C Pembrolizumab combined with GDP regimen inducing sustained remission in histiocytic sarcoma: A case report. J Investig Med High Impact Case Rep 2024; 12: 23247096241274561. 10.1177/23247096241274561 PMC11366101 39215504

[13] Nguyen LT Pham GH Vu PT Favorable outcome of a histiocytic sarcoma patient treated with immune checkpoint inhibitor: a case report. Ann Med Surg (Lond) 2023; 85: 6274–8. 38098600 10.1097/MS9.0000000000001446 PMC10718375

[14] Ruan GJ Zanwar S Ravindran A Clinical characteristics, molecular aberrations, treatments, and outcomes of malignant histiocytosis. Am J Hematol 2024; 99: 871–9. 38409747 10.1002/ajh.27263 PMC11038892

